# Ownership of individual-level health data, data sharing, and data governance

**DOI:** 10.1186/s12910-022-00848-y

**Published:** 2022-10-29

**Authors:** Jan Piasecki, Phaik Yeong Cheah

**Affiliations:** 1 Department of Philosophy and Bioethics, Faculty of Health Sciences, Jagiellonian University Medical College, Kopernika 40, Kraków, Poland; 2 Mahidol Oxford Tropical Medicine Research Unit (MORU), Faculty of Tropical Medicine, Mahidol University, 420/6 Rajvithi Road, Bangkok, Thailand; 3 Centre for Tropical Medicine and Global Health, Nuffield Department of Clinical Medicine, University of Oxford, Old Road Campus, Roosevelt Drive, Oxford, UK; 4 The Ethox Centre, Nuffield Department of Population Health, University of Oxford, Oxford, UK

**Keywords:** Data ownership, Individual-level health data, Data access committees, Sharing data, Clinical trials, Research ethics, Learning healthcare system, Embedded research, Ethics

## Abstract

**Background:**

The ownership status of individual-level health data affects the manner in which it is used. In this paper we analyze two competing models of the ownership status of the data discussed in the literature recently: private ownership and public ownership.

**Main body:**

In this paper we describe the limitations of these two models of data ownership with respect to individual-level health data, in particular in terms of ethical principles of justice and autonomy, risk mitigation, as well as technological, economic, and conceptual issues. We argue that undifferentiated application of neither private ownership nor public ownership will allow us to resolve all the problems associated with effective, equitable, and ethical use of data. We suggest that, instead of focusing on data ownership, we should focus on the institutional and procedural aspects of data governance, such as using Data Access Committees (DACs) or equivalent managed access processes, which can balance the elements of these two ownership frameworks.

**Conclusion:**

Undifferentiated application of the ownership concept (private or public) is not helpful in resolving problems associated with sharing individual-level health data. DACs or equivalent managed access processes should be an integral part of data governance. They can approve or disapprove data access requests after considering the potential benefits and harms to data subjects, their communities, primary researchers, and the wider society.

## Background

### Data ownership

The ownership status of individual-level health data could affect biomedical research, individual freedoms, and the free-market economy [1–5]. Ownership of a certain thing determines the actual ability or legal right to exclude others from using the thing in question [3, 4]. Therefore, it is unsurprising that, despite the existing detailed regulations in most developed countries, the concept of data ownership is a subject of ongoing debate [5].

A system of health data governance must balance the interests of multiple stakeholders (data subjects, researchers, sponsors, and society) as well as overcome technological, economic, legal, and ethical challenges. As a result, data governance systems are complex and consist of technological solutions and bureaucratic procedures. For an individual data subject, the upshot is confusion and lack of clarity in regard to the scope of data control.

In this paper we focus on the concept of *data ownership*. The concept of ownership—as it is argued by some representatives of both sides in this debate—is key in resolving ethical and practical controversies surrounding access to health data. On the one hand, there are those who want to privatize data (Privatization Postulate, PP): for them, *private ownership* is construed as a means of individual control over one’s data, privacy, and property [6–9]. On the other hand, advocates of open science argue that individual-level health data should be a common good [1, 3, 4]. This trend is referred to as the Communization Postulate (CP) or *public ownership*.

We argue that undifferentiated application of the ownership concept (private or public) is not helpful in navigating practical and ethical challenges of access to individual-level health data. Both postulates (PP and CP) may seem appealing—they refer to basic moral principles such as autonomy and justice and to the seemingly intuitive concept of ownership. They appear to be a simple solution to a complex problem, but they pose practical, theoretical, and ethical problems. The debate between these two opposite views (undifferentiated *private* and undifferentiated *public data ownership*) diverts public attention from alternative ideas which apply a more nuanced approach to ownership and have been discussed in the literature recently, such as data cooperatives, public data trusts, good data sharing practices, and the idea of personal data sovereignty [10–12], as well as from devising effective data governance systems that would allow tackling the challenges of new technologies, research, and learning healthcare systems (LHS). We use the example of Data Access Committees (DACs) to show how such a data governance body can resolve some of the challenges of data sharing [13]. We argue that DACs play a role of a safeguard protecting individual and communal interests. However, advocating in favor of DACs allows us to demonstrate the ethical and practical relevance of a nuanced approach to data ownership in this context.

## Terminological clarification

The terminology of the debate over health data is both technical and ambiguous. Therefore, we begin with a terminological clarification, and we wish to emphasize that our arguments and conclusions are not necessarily generalizable beyond the scope delineated here.

The term “individual-level health data” refers to data generated in clinical research or clinical practice (e.g., electronic health record or EHR). We do not refer to any other kind of “health data” that is derived from social media (e.g., Twitter), wearable technologies (e.g., smartwatches), patient sites (e.g., Patientlikeme.com), data from web browsers and Internet search engines, or aggregated data from research projects, etc.

Individual-level health data should be distinguished from individual records held in certain physical carriers of information. In this discussion, we are referring to the former. We assume that when the proponents of the PP argue that an individual has a legally enforceable claim to her individual data, they mean that the individual has a right to have her data erased from physical drivers belonging to a private company or public institution. When the adherents of the CP argue that a government or its agencies own certain elements of individual health data, this means that a private company or research consortium is obliged to either deliver copies of certain records to a public repository or give access to this data on its own servers.

The term “data governance system” refers to a system which governs access to individual data. Individual data is data that pertains to a physical, identifiable person. In such a system an individual has certain rights, e.g., to limit access to her data or to grant access to some parties (authorize them). Such a system of data governance can be legally defined, for instance by regulations such as the European Union General Data Protection Regulation. However, we do not refer to any legal solution here. In such a system DACs or equivalent managed access processes can play an additional role, guarding interests of individual research participants/patients, research institutions, and scientific integrity in general. Both the PP and CP would entail changing the main conceptual approach of the existing data governance systems.

In this paper we focus on individual-level health data that can be de-identified and shared. De-identification is a process in which typical direct identifiers, such as first and last name, phone number, insurance number, and any kind of identification numbers, are stripped from data in order to preclude identification of the data subject. However, de-identification cannot be seen as a process that allows the legal and ethical commitments of healthcare professionals and researchers to be circumvented. Data sharing usually involves de-identified data within the constraints of previous (broad) informed consent or other legal requirements (e.g., impracticality of reconsent and additional ethics review).

## Different kinds of good and normative arguments

Intuitively, most people know what it means to own something. If one is an owner of a certain thing, one has that thing at one’s disposal. The concept of ownership grasps two elements of individual control over things: factual and normative. In a factual sense, if one is an owner of something, one can control that thing, e.g., a bicycle. It would be an example of senseless or metaphoric use of language to say that one owns a star, which cannot be controlled. In a normative sense, if one is an owner of a certain thing, then others are prohibited from using that thing without one’s permission, otherwise one’s property rights are violated.

However, there is more than just one concept of ownership. For instance, from a legal perspective, ownership can be divided and different people can have different aspects of an object at their disposal. Person A can be an owner of a certain property, but person B can have a right to live on this property until the end of her life. It means that A’s actions are restricted by B’s rights, and even when A sells the property to C, B still keeps the right to live on that property. An example of an even more complicated ownership concept involves shares in companies, royalty rights, etc.

The discussion over health data ownership usually employs the economic concept of ownership [3, 4, 14]. The economic concept is devised to grasp people’s relationship with more complicated material and non-material objects that can be owned, such as books, seas, streets, and parks. This concept of ownership can describe not only individual, but also group ownership. Therefore, economists distinguish at least four different categories of goods or items that can in a certain sense be owned: i. private goods; ii. public goods, iii. club goods, and iv. common pool resources [3, 4, 14]. According to this classification, every good is characterized by two features regarding its accessibility: excludability and rivalry (see Table 1). A good is excludable when only one person can effectively stop another person from using or consuming it (e.g., a berry). A good is not excludable when a person cannot effectively stop another person from enjoying it (e.g., fresh air). A good is rivalrous when a person consuming that good subtracts from the pool available to others (e.g., admission at a public university). A good is not rivalrous when a person who consumes it does not subtract from the pool (e.g., public security).

**Table 1:** Type of goods

	Private goods	Club goods	Common-pool resources	Public goods
Excludability This means that a certain thing (good) is mine I am in a position to exclude others from using it. For instance, I hold onto my pencil and do not allow others to use it. But I cannot appropriate sunny weather just for myself. Sunny weather is a non-excludable good	Yes	Yes	No	No
Rivalry/Subtractability This means that when I use a thing, I am taking a part of that thing from others. When I eat a berry there are fewer berries for others. But when I enjoy safe traffic in my city I do not diminish the pool of safety for others. Safe traffic is a non-rivalrous good	Yes	No	Yes	No
Example	A pencil	A membership- based botanical garden	Berries in the public woods	Public security

However, the question “to what category of goods—private, public, club, or common pool resources—should individual-level health data belong?” is not purely based in the characteristic of that particular good. We can try to flip certain items from one category to another using laws or other formal or informal social contracts and arrangements. Generally speaking, we can communize or privatize certain items. For instance, in communist regimes formerly privately owned means of productions, e.g., factories, were communized. When the regime changed, they were again privatized. Communization of means of production could be motivated morally and ideologically, but it may fail economically. In turn, privatization of factories may have good economic consequences in the long run but can be perceived as morally unacceptable if it causes unemployment and impoverishment of a certain group. This example demonstrates that in the discussion on what category certain good should be assigned to various arguments are used: ethical, practical, and economic. However, as we see, economic debate can prompt political and legislative action. The main problem of both the PP and the CP postulates is their undifferentiated approach to the legal concept of ownership. In the case of the PP, an individual has an indivisible property right to her data; the same is true in the case of the CP, but the property right is bestowed to the public. Nevertheless, as we try to demonstrate, a more nuanced approach is needed to balance the interests of all stakeholders.

In the discussion about the right assignment of individual-level health data debaters refer also to different kinds of arguments. Some of them will refer to the characteristics of the thing in question. For instance, one can argue that data cannot be easily appropriated because it can be copied and stored on multiple data carriers. Others, on the contrary, will be using normative arguments that refer to possible ethical consequences of certain *ownership* laws. A normative argument could be the following: “although it is hard to control one’s data, an individual has a moral right to control her individual-level health data”.

We also follow the same argumentative strategy: we try to arrive at a normative conclusion, though our argumentation also uses descriptive arguments and refers to some intrinsic characteristics of individual-level health data.

In the following paragraphs we argue that the PP and CP face similar challenges: 1. Justice problem: how to properly balance individual and public interests; 2. Autonomy problem: how to respect data subjects’ autonomy; 3. Risk mitigation problem: how to protect individual or/and institutional interests; 4. Practical and conceptual problems.

## The privatization postulate (PP) and its problems

There are two main ethical arguments in favor of the PP: the first uses utilitarian calculus of possible benefits, while the other makes a reference to the principle of autonomy. However, both are dubious, and the whole model faces more challenges, as demonstrated in the forthcoming sections.

The first argument pertaining to public good and utilitarian calculus is that privatization of data creates incentives for efficient use of data. Proponents of this option offer a ready-made solution [6–9]. If individuals, as rational and economic creatures, and private companies driven by the creation of profit have an opportunity to make money from data, they will. In this instance the invisible hand of the market would contribute to the development of science and public health: individuals and companies will aim to generate profits and the creation of scientific knowledge will be a byproduct. Private data ownership is then a key consideration, because when no party can profit from data, they are not used. Health data are derived from an encounter between an individual patient or a research participant and a health care provider or researcher [6]. Both have stakes in the data obtained from this encounter. A middleman, e.g. an independent databank tasked with collecting individual data, ensuring informed consent and privacy regulations, can make the data work for the patient and for the greater good [6]. Such an institution would profit from selling access to data and share the benefits between all three parties involved: the patient, the health researcher/institution, and itself. This would also help to control access to individual-level health information in the long term [6–9].

The second argument is that property is an effective tool of exercising one’s autonomy and protecting one’s privacy. This means an individual can sell or give her data only to a party she chooses. The individual also has the right not to sell or give her data at all. However, there are some problems with the PP that are discussed in the following sections.

### Justice and privatization

The PP does not provide a reliable instrument to balance individual and public health interests. The proponents of the PP argue that privatization of individual-level health data will create a circle of supply and demand of data, and therefore the invisible hand of the market will take care of the rest: researchers will get the data, and individuals and middle-men will get their money. The problem is that market demands are not always congruent with public health needs and requirements of justice. The invisible hand of the market cannot be the only instrument to address these issues. For instance, vulnerable populations might be underrepresented in private databanks, due to medical illiteracy or having lack of contact with healthcare services. Moreover, certain rare conditions would require pooling data from different parts of the world without a promise of sufficient income for private companies [15].

Besides that, privatization of data would create additional financial barriers for data sharing, instead of creating incentives. Secondary data users such as research institutions would have to pay additional charges for access to data. Meanwhile, one can argue that sharing data for developing science and health care might be considered a contribution to the public good [16]. In wealthy states, which provide their citizens with access to healthcare, everyone enjoys advances in the health sciences and it is virtually impossible to exclude anyone from benefiting from this good. Industrialized nations build a scientific infrastructure and the society as a whole invests in health sciences. Even private companies use public funds and public infrastructure to conduct their studies. The principle of justice requires solidarity and reciprocity. It entails that if one enjoys the result of scientific progress, one is also obliged to demonstrate reciprocity and solidarity with those whom science has not yet benefited. One way to reciprocate the benefits of progress is to make a contribution to the development of science by making individual-level health data available for research. Therefore, we can justify making de-identified individual-level health data available with reference to justice, and privatization of data would violate the justice principle in that sense. However, one can claim that the arguments referring to reciprocity and justice do not undermine private ownership as such, but they rather point out moral obligation to make one’s data accessible. As discussed in Sect. 4.4, counting on volunteers in data research is not sufficient to collect data that is representative.

### Autonomy and privatization

Barbara Evans points out that those who demand their data property rights seem to expect that these rights have an absolute status and that, in every circumstance, they can refuse to give access to their property [2]. This is not true, as in many jurisdictions, a state has the power to access private property or to even take it away. Since governments have appropriation power guided by social interests, there is no reason to assume that an existing legal framework protects privacy less than a future property law would [2]. In a hypothetical situation where data belongs to individuals who do not want to share it for research conducted for public interest, this data, in theory, can be legally taken by a government or its agencies. The justification and the scope of this data harvesting would resemble a situation that can occur under existing regulations. However, Evan’s argument refers only to a situation where a government is interested in individual-level health data, and it does not involve cases.

Moreover, if a patient or participant is to fully control her data, the *private ownership* model requires a middleman databank that only rents access to her data. Otherwise, when data is sold, she does not have control over it once sold, data can be resold to other parties. Therefore, it seems that despite *private ownership* individual control is not absolute regarding government appropriation and requires robust third-party databanks in order to maintain control over one’s data.

### Risk and privatization

The *private ownership* model poses some problems to embedded research. We have defined “embedded research” as research which acquires data in the process of providing health care, e.g., capturing data from patients’ EHRs, and then analyzes the data with the intention to improve future care. This closed cycle of collecting data, analysis, and care improvement is the essence of LHS [17]. One important feature of LHS is to identify overperforming providers and copy their good practices [17]. In order to do this, all individual-level health data form all healthcare system institutions is needed.

The main goal of the *private ownership* model is to give the right to control the data to individuals, not to institutions. It means that an individual has the right to give or sell her data to her own healthcare provider or third parties e.g. other institutions or databanks. Without a full dataset, embedded research cannot be conducted appropriately and wrong conclusions may be made about a provider’s performance. Moreover, these wrong conclusions if they are negative, can result in reputational risks and loss of trust by patients in their healthcare provider.

Therefore, a more flexible approach is needed. A data governance model can prevent this problem and gives some reasonable means of preventing reputational risks to institutional healthcare providers.

### Practical, conceptual problems and privatization

There are further questions about what the *private ownership* model would look like in practice. If the *private ownership* model really limits access to individual-level health data in a healthcare system, then from a scientific and methodological point of view, epidemiological and public health research, for which informed consent is usually waived, could be stymied, as it would not allow researchers to collect representative samples of participants and their research would lack statistical relevance. It has been proven that opt-in studies are not representative and create biased samples [18].

Moreover, there is an economic issue, although we acknowledge that we do not have empirical data, hence the arguments of both sides are speculative. The proponents of the PP argue that private ownership of data does not impede research based on individual-level health data. On the contrary, they claim that private ownership will boost research and economy [6–9]. The plan is simple: individuals can sell rights to use their data to a middleman, a private health information bank, which will then sell the data to researchers. And yet, is this a viable solution from an economic point of view? The argument is that this solution does not necessarily generate sufficient economic gain to cover all cost. It advertises itself as economically self-sustainable, which does not need governmental financial incentives that usually go with governmental paternalism. Evans argues that investment in data infrastructure and data curation may be economically unsustainable [2]. We can speculate that even if this solution is feasible from an economic perspective, the ramifications seem to include increasing fees for researchers, who would have to cover the costs of data preparation, infrastructure maintenance, and the revenues of middlemen and data owners, and, as a consequence, higher costs of research for the whole society.

## The communization postulate (CP) and its problems

The CP is supported not only by a moral argument that points to the profits for the healthcare community, but also by an economic one, which considers knowledge (and *ipso facto* data) to be a special kind of good that should be accessible to all [14]. This argument could be even more compelling if one agrees and sympathizes with the trend toward open research and transparency in research [19].

The CP regarding individual-level health data entails creation of a system that would allow cultivating the commons of data infrastructure. This is a system of common practices that protect and incentivize the production of common goods and put enforceable norms in place to impose some level of conformity. The supporters of this view believe that a manageable system of commons should provide all parties involved with information that allows them to make informed decisions and have a system of conflict resolution, enforcing compliance with accepted norms. Necessary infrastructure is a part of this system, which as a whole has to be adaptive to technological changes in storing, processing, and transmitting information [14]. Having all these elements in place should secure a sustainable common pool of resources: scientific knowledge. This system is currently being built in open-access journals, public libraries, national and international research centers, and research programs.

It seems reasonable to postulate that de-identified individual-level health data could become part of this system in open repositories. There are, however, a few problems, which are discussed in the following sections.

### Justice and communization

Communization of individual-level health data seems to resolve the problem of health data accessibility. Since all data that exists is common, every researcher or secondary user is entitled to access it. Focused on one aspect of data accessibility, namely: possible exclusion of some parties from its use [20]. However, it does not address the problem of data supply. Thus, the framework of commons does not adequately capture the ethical problems of power asymmetry and inequalities with respect to data accessibility [20]. We concur with Prainsack that the problem of equitable access to data is not limited to secondary uses, but it must address the problem of data supply, especially data from vulnerable and underprivileged populations. Vulnerable and marginalized communities can only benefit from research when their data is included in the data pool.

Moreover, equitable inclusion entails engagement with populations in the process of decision making, data governance, and benefit sharing. These issues are not addressed by the conceptual frameworks of commons. Therefore, it appears that in this context, the CP faces the same ethical challenge as the PP: there is no proper institutional and procedural instrument that would allow promoting research among underprivileged groups and balancing individual and public interests.

### Autonomy and communization

The collection and processing of data in a clinical care, research or for a LHS involves an agreement between researchers/healthcare staff and participants/patients. Such an agreement specifies the scope of the data collected, its processing, safeguards, retention, the purpose of data processing, and sharing practices. The agreement is ethically and, in some jurisdictions, legally binding even when data is de-identified. This means that if a participant agrees to share her data for diabetes research, she does not want her data to be used in research on other diseases such as mental disorders or fertility. Even if the data were de-identified, researchers are obliged not to share her data with a research project searching for links between diabetes and fertility.

The central problem with the CP is that it does not recognize the agreements between researchers and participants. In this model, de-identified individual-level health data is a public good and can be used freely in the public domain.

It seems that the CP entails overly lax access to data that could undermine trust between healthcare professionals, researchers, and the public. There is evidence that some patients are afraid to share all their information with healthcare professionals [21]. If a significant number of patients do not share important health information with healthcare professionals, then it could affect not only embedded research, but also public health.

A data commons theorist could revoke the common practice in public health and epidemiological research of waiving requirements of informed consent and accessing individual health data on the basis of public interest coupled with difficulties in obtaining consent from a large group of participants. However, this practice does not prove that individual health data should be treated as a common good, but rather that individual rights can sometimes be trumped by public benefits that cannot be achieved otherwise.

Another way of defending the CP’s position would be to nuance it by demanding that only some types of data should be considered as commons, and sensitive data would not be present in the public domain. Nuancing the CP makes this position more similar to the already existing solution that allows circumventing informed consent requirements in certain circumstances. However, it does not fully address the problem of autonomy that we have discussed above and it does not give a patient or participant a personal choice to make only some of her data accessible to researchers or specify the type of permitted uses.

### Risk and communization

The use of individual-level health data carries risks. The process of de-identifying individual-level health data does not entirely protect individuals and groups from all possible risks [22]. One risk to individuals is re-identification [23–26]. If de-identified individual-level health data is coupled with publicly available data (for instance, voter registration databases in the US), then the identity of the data subject can be revealed [24]. But the breach of individual privacy and its consequences (e.g., financial loss, loss of insurance and access to healthcare) are not the only risks. There are also group- risks that affect not only individuals, but also stigmatized and discriminated groups. Moreover, health data can have different levels of sensitivity [27]. Sensitive health data can contain information about mental disorders, sexually transmitted diseases, fertility problems, facial and body images, and genes. Therefore, it seems reasonable to limit and control access to individual-level health data, even if they are de-identified at the individual level, rather than treat it as a common-pool resource or a public good.

In Sect. 4.3 we discussed possible risks to institutional healthcare providers that are associated with embedded research. The same problem exists with the *public ownership* model. In such a model, institutions would also have no proper instruments to protect their interests.

The CP does not have any instrument to address the issue of data sensitivity, because all data—when de-identified—is common. The data governance model, which will be discussed in Sect. 6, allows us to impose special protection on sensitive data.

### Practical, conceptual problems and communization

The CP proponents argue that infrastructure for commons of individual-level health data should be created to make it accessible to everyone who wants to carry out research and to advance public interest.

There are at least two problems with the CP in this regard. Firstly, it is not clear who will bear the costs of such transformation and maintenance and curation of such a system. The second problem is closely associated with the first: even if individual-level health data belong in the public domain, we do not know who the owner of the infrastructure should be. It seems that the system can only be stable when the state is the owner of the infrastructure. Otherwise, a private owner of the server would have a right to shut down the business and destroy the property. But the state is not necessarily interested in all kinds of data, because not all kinds of individual-level health data can advance public interests. It means that the CP would limit itself only to certain kinds of data that are important for public health and can advance public interest. In that case, the CP would be to some degree consistent with the existing systems of data governance and would coexist with the other systems of data governance. These other systems are necessary to govern data that are not of interest by governments, but are important in biomedical research and advancement of science.

## Governance of data and the role of the DACs

We have outlined the limitations of the two models of ownership, i.e., public and private, with respect to individual-level health data that can be de-identified for secondary uses. We have argued that neither the PP nor the CP model is appropriate in the context of sharing individual-level health research data. However, we think that a governance framework for managing access to de-identified individual data should include combined elements of these two models. Data should be protected to some extent to protect individual privacy and to protect incentives for conducting research that involves primary data collection. On the other hand, data could be treated as a public good or a common-pool resource if there is a proper governance structure in place. The tragedies of commons and anticommons are preventable by proper governance; for example, secondary users’ requests to use datasets can be channeled via a Data Access Committee or an equivalent managed access process.

A DAC can balance the elements of these two frameworks. In that sense, when we advocate for DACs’ role in data governance, we refer to the ethical premises: a DAC is a structure that protects individual rights by sharing data only within the scope of informed consent obtained from data subjects apart from in exceptional circumstances. It promotes use of data in the public interest. It also solves the problem of ownership of data from research conducted by multiple institutions. A DAC does not own or store the data; it acts as a custodian of datasets. Moreover, DACs should not be confused with databanks that manage the data on behalf of individual data owners—as discussed earlier. A DAC can be institutional, such as the Mahidol Oxford Tropical Medicine Research Unit DAC [28], independent, such as the Infectious Disease Data Observatory DAC [29], or act on behalf of a large research group or consortium, such as the MalariaGEN DAC [30]. If the DAC acts on behalf of a research group, it has the added responsibility of balancing the interests of various institutions.

A DAC reviews the reasons and the credentials of applicants for secondary use. Many secondary users are researchers studying the same disease or topic, and this could directly benefit the data subjects and their communities. Data can also be requested for scrutiny by researchers external to the primary research group for independent review. While the former has direct benefits, the latter promotes trust in the scientific enterprise. Governments and public health institutions also have legitimate reasons for obtaining access to individual health data, for example, to allocate health resources within a country or to curb the spread of a pandemic. In these cases, for justice and reciprocity reasons, individuals are unlikely to be able to opt out of contributing their data.

To mitigate the risks of data sharing, permission will not be granted for data reuse that could potentially put participants at risk of harm, such as discrimination or stigmatization. This can be achieved if DACs have members who understand the communities from which the data is collected or have ongoing engagement with those communities. In addition, data access agreements will need to be put in place to ensure that data requesters do not use data for reasons other than those stated, that they do not try to identify the participants, and that they have adequate infrastructure and procedures to protect the confidentiality of participants.

An important role of a DAC is to promote equitable access of data. If data is completely open access, researchers in low-resource settings who lack excludable goods such as money, equipment, and expertise will be at a disadvantage. Indeed, there has been a lack of requests for data access by researchers from low-resource settings [28]. This could exacerbate the existing inequalities between secondary users in high- and low-resource settings. Researchers and others working in well-funded institutions can analyze and publish papers much more quickly than those in resource-starved institutions. They could then use these academic outputs attain further funding and academic accolades. The upshot then resembles colonial economy: the developing countries provide the developed ones with data (raw material), which is then processed by scientists equipped with highly specialized hardware and software, who also profit the most from this state of affairs by producing scientific publications.

To mitigate this problem, some conditions could be put in place for sharing data generated in low-resource settings. These could include requirements for benefit sharing, capacity building and collaborations. For example, a local investigator or data scientist could be included in the research team conducting the secondary analyses and be included as an author in any resulting outputs. Priority could be given to secondary analyses with research questions that directly address the health problems of communities that generate the primary data [31]. In this sense, a DAC is an instrument that allows meeting the requirements of justice.

DACs will also ensure that the shared data does not contain any personally identifiable information, and that data is used within the scope of the broad consent provided by patients or research participants. This guarantees that participants have control over how their data is used in the future. This approach makes it possible to respect both the wishes of the participant with regard to future use and the altruistic reasons for participating in research and contributing their data in the first place.

As mentioned above, the PP and CP can be appealing regulatory solutions because they both provide us with simple and almost automatic solutions for data accessibility. In the PP, no one is in charge, and everything is somehow managed by market forces. The CP seems to promise free access for everyone. But as we have demonstrated throughout this paper, the data governance system needs to balance multiple interests: individuals, communities, primary data collectors, secondary data users, editors, research funders, and research sponsors. Moreover, data should not only be protected, but the use of data should be promoted for the public benefit. Therefore, the ethical argument in favor of a managed access system including the use of a DAC is prudential: at least thus far, it seems to be the best approach for balancing multiple ethical values and interests and it seems to avoid the problems associated with privatization and communization of individual-level health data.

## Conclusions

In conclusion, it can be said that undifferentiated application of the ownership concept (private or public) is not helpful in resolving problems associated with sharing individual-level health data. Different parties have rightful stakes in data: individuals, society as a whole, governments, private sponsors, researchers, and research communities. This means that undifferentiated application of the ownership concept, whether private or public, is insufficient to balance the interests of all data stakeholders. We have to carefully balance the different rightful claims from different data stakeholders and keep adjusting our regulations to the changing technological and economic environment. DACs or an equivalent managed access system are one element of a complex system of data governance; important questions about public trust, data capitalism, and individual freedoms remain to be answered.

## Data Availability

Not applicable.

## Abbreviations

CP: Communization postulate
DAC: Data access committee
EHR: Electronic health record
LHS: Learning healthcare system
PP: Privatization postulate
